# An efficient protocol to generate placental chorionic plate-derived mesenchymal stem cells with superior proliferative and immunomodulatory properties

**DOI:** 10.1186/s13287-019-1405-8

**Published:** 2019-10-17

**Authors:** Qilin Huang, Yi Yang, Chen Luo, Yi Wen, Ruohong Liu, Shuai Li, Tao Chen, Hongyu Sun, Lijun Tang

**Affiliations:** 1 Department of General Surgery & Pancreatic Injury and Repair Key Laboratory of Sichuan Province, The General Hospital of Western Theater Command (Chengdu Military General Hospital), Chengdu, 610083 China; 2 College of Medicine, Southwest Jiaotong University, Chengdu, 610031 China

**Keywords:** Mesenchymal stem cells, Isolation, The modified explant culture method, Chorionic plate-derived MSCs, Macrophage

## Abstract

**Background:**

Placenta-derived MSCs (P-MSCs) represent a promising tool for cell-based therapeutic applications. However, the increasing demand for P-MSCs in clinical trials makes high quality and large number of P-MSCs mandatory. Here, we aim to develop an efficient protocol for P-MSC isolation and culture.

**Methods:**

The modified explant culture (MEC) method by combining an initial mild enzymatic reaction with the subsequent explant culture was developed to simultaneously produce various P-MSCs from the different regions of the placenta in serum-free medium (SFM). Its isolation efficiencies, cell yield, and proliferative capacity were compared with the conventional explant culture (EC) method. Furthermore, we determined whether functional properties of P-MSCs are affected by the used tissue-harvesting sites in terms of their proliferation, migration, and the immunomodulatory effect on macrophage.

**Results:**

The MEC method achieved higher yield and shorter time in primary cell confluence in SFM compared with the conventional method. The harvested cells possessed the MSC characteristics and demonstrated significantly stronger proliferation ability. Importantly, MSCs derived from chorionic plate (CP-MSCs) were found to exhibit superior properties to the other P-MSCs in proliferation and migration capacity, maintaining the fetal origin over serial passages. Notably, CP-MSCs show stronger ability in regulating macrophage polarization from M1 to M2.

**Conclusion:**

Our study developed an efficient and high-yield technique to produce high-quality P-MSCs from the placenta, hence serving as an optimal source of MSCs for clinical application.

## Background

Mesenchymal stem cells (MSCs) are considered as potential therapeutic tools for clinical application due to their beneficial characteristics including high self-renewal and multipotent differentiation [1], low immunogenicity, and immunomodulatory abilities [2, 3]. Accumulating evidence from clinical trials indicates that MSCs exert therapeutic effects in numerous diseases such as graft versus host disease (GVHD) [4], liver failure [5], acute myocardial infarction [6], rheumatoid arthritis [7], and liver cirrhosis [8]. Originally, MSCs were isolated from the bone marrow (BM). However, BM is not an ideal source of MSCs in view of the low cell numbers, the invasive harvesting procedure, and the reduced proliferation capacity by age [9–11]. Therefore, it is necessary to find out alternative MSC sources for clinical applications.

Perinatal tissues like the placenta and umbilical cord are promising MSC sources because they possess several advantages such as easy accessibility, noninvasive procedures, and minimal ethical constraints. Compared to MSCs derived from adult BM or adipose tissues, placenta-derived MSCs (P-MSCs) and umbilical cord-derived MSCs (UC-MSCs) are generally considered as fetal cells with superior proliferation ability [12], stronger immunomodulatory [13], and lower immunogenicity [14]. Moreover, a recent study found that P-MSCs possess better immunoregulatory properties compared to UC-MSCs [15]. Hence, it seems that the placenta is the better choice for obtaining MSCs. Currently, various kinds of P-MSCs have been successively isolated from different anatomical regions of the placenta, including chorionic plate-derived MSCs (CP-MSCs), chorionic villi-derived MSCs (CV-MSCs), amniotic membrane-derived MSCs (AM-MSCs), and decidua-derived MSCs (D-MSCs). However, there is no consensus on the quality of the current cultured P-MSCs, thereby making standardization production of P-MSCs difficult. As reported in the literature, the current cultured P-MSCs are usually confounded by maternal cell contamination [16–18]. The reason behind this phenomenon is complicated, like the variation in MSCs obtained from different regions of the placenta, culture system, and so on. Therefore, it is mandatory to define the key parameters to obtain high-quality MSCs like as pure fetal P-MSCs for clinical trials.

As P-MSCs are gaining more and more attention in clinical trials, there are increasing concerns regarding the isolation and culture. However, no standard isolation methods for P-MSCs are available. Currently, the main methods for isolating P-MSCs are the enzymatic method and the explant culture (EC) method. In the enzymatic method, the tissue block is digested with proteolytic enzymes, and the cell suspension obtained is cultured. However, some studies have demonstrated that the enzymatic method affects the biological characteristics of MSCs such as proliferative capacity and reduces cell viability due to prolonged digestion [19, 20]. Although the MSCs obtained by EC have better biological functions, it takes longer time to reach cell confluence due to the slower migration of cells from the tissue block. Therefore, it is necessary to develop a simple and efficient method to obtain larger numbers of P-MSCs in a shorter time.

In this context, our study aims to develop and optimize an efficient isolation and culture system for harvesting large numbers of pure fetal P-MSCs with superior properties. The modified explant culture (MEC) method by combining an initial mild enzymatic reaction with the subsequent explant culture was developed to simultaneously produce various P-MSCs from the different regions of the placenta in serum-free medium (SFM). Its isolation efficiencies, cell yield, and proliferative capacity were compared with the conventional explant culture (EC) method. Moreover, the properties of various P-MSCs were also compared in terms of their origin, proliferation, migration, and the immunomodulatory effect on macrophage.

## Methods

### Sample collection

Approval of the Institutional Ethics Committee of the General Hospital of Western Theater Command was obtained for the sample collection, processing, and other experimental procedures before initiating this experiment. Human placentas of uncomplicated, elective cesarean deliveries at term were collected after obtaining written informed consent from the mother. And only pregnancies with a male fetus were chosen.

### Anatomical dissection approach

**Fig. 1:**
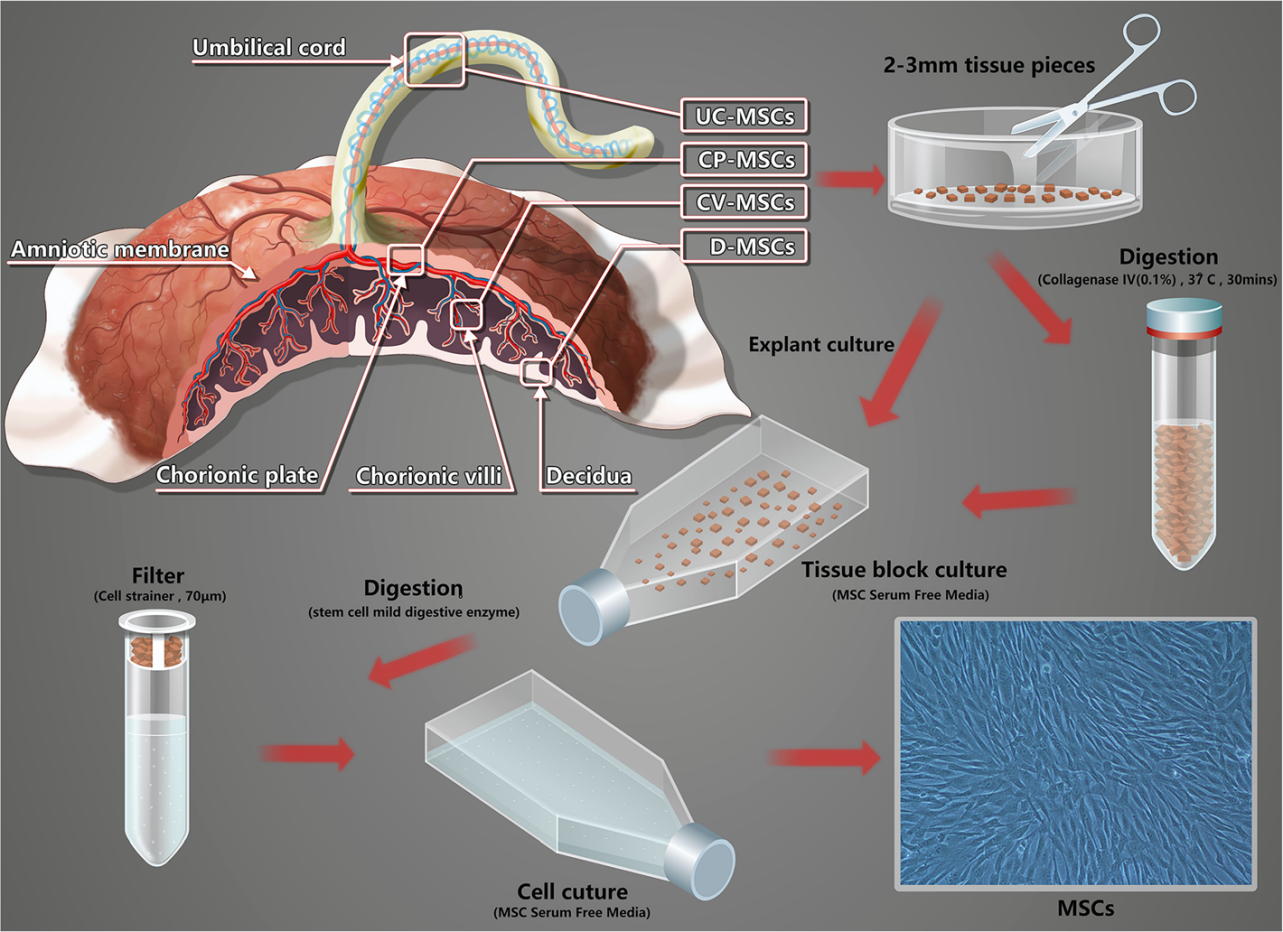
Schematic diagram of the isolation of MSCs from the human umbilical cord and placenta. MSCs were isolated by MEC and EC from the different regions of the placenta and umbilical cord. Modified explant culture (MEC): the shredded tissue block was transferred to a centrifuge tube and incubated with digest media (0.1% collagenase IV) for 30 min, at 37 °C with gentle rocking. After digestion, tissue block was washed three times with PBS, centrifuged (300*g*, 5 min, 4 °C), resuspended with MSC Serum Free Media, then transferred to a T75 culture flask and cultured. Explant culture (EC): the shredded tissue block was mixed with MSC Serum Free Media, and then transferred to a T75 culture flask and cultured

MSCs were isolated as described [16, 21–23] but with modifications to select placental tissue based on anatomical structure (see Fig. 1).

#### Umbilical cord-derived (UC)-MSCs

The umbilical cord tissue was cut and then placed in a sterile petri dish with wash of phosphate-buffered saline (PBS) containing 1% penicillin-streptomycin (Gibco, USA) to remove the blood on it. Finally, the umbilical cord tissue was minced.

#### Chorionic plate-derived (CP)-MSCs

Upon removal of the amniotic membrane from the fetal surface of the placenta, the chorionic plate from the region closest to the umbilical cord was dissected, then washed and minced.

#### Chorionic villi-derived (CV)-MSCs

Approximately 0.5 cm deep into the placental tissue was dissected from chorionic villi from the region closest to the umbilical cord and ≥ 1 cm away from the maternal side of the placenta (decidua). Chorionic villi tissue was placed in a sterile culture dish, washed, minced, digested, and inoculated to a T75 culture flask.

#### Decidua-derived (D)-MSCs

After the maternal side of the placenta was faced upward, decidua tissue was cut from the center of the placenta with the cutting depth controlled within 0.5 cm by a sterile anatomical instrument. The decidua tissue was placed in a sterile culture dish, then washed, minced, digested, and inoculated to a T75 culture flask.

### MSC serum-free culture system

MSCs were isolated as early as possible after delivery of the placenta, commonly within 3–6 h. According to the placental anatomy, cut the appropriate amount of tissue blocks from the corresponding parts of the placenta and umbilical cord, and then transfer the tissue blocks to a sterile culture dish with wash of PBS containing 1% penicillin-streptomycin. The tissue block was cut with a sterile tissue scissors (2–3 mm in diameter) and then divided into two portions, one for MEC and the other for EC. (A) Modified explant culture (MEC): the shredded tissue block was transferred to a centrifuge tube, and then incubated with digest media (serum-free Dulbecco’s modified Eagle medium (DMEM; Hyclone, USA) containing 0.1% collagenase IV (Sigma, USA)) for 30 min, at 37 °C with gentle rocking. Freshly prepared digest media was incubated at least a 1:1 ratio with the tissue block. After digestion, tissue block was washed three times with PBS and then centrifuged (300*g*, 5 min, 4 °C). The pellet was resuspended with MSC Serum Free Media (Yocon, China), and then transferred to a T75 culture flask and cultured at 37 °C in a humidified atmosphere with 5% CO2. (B) Explant culture (EC): the shredded tissue block was mixed with MSC Serum Free Media, and then transferred to a T75 culture flask and cultured at 37 °C in a humidified atmosphere with 5% CO2. (Note: do not move the T75 culture flask for the first 3 days). From the fourth day, the state of cell growth was observed every 2 days, and subculture was carried out at a density of 8000/cm^2^ when primary cell confluence reaching approximately 80%. Detailed method of MSC subculture is seen in Additional file 1.

### Macrophage and MSC noncontact co-culture

Macrophages were isolated as described earlier, and the detailed method is seen in Additional file 1 [24]. First, macrophages were treated with 1μg/ml lipopolysaccharide (LPS; sigma, USA) for 24 h to induce polarization to M1, and then macrophages and MSCs were co-cultured at 5:1 through transwell chamber (0.4-μm pore size; Corning, USA) for 24 h, where macrophages and MSCs were located in the lower and upper compartment of the chamber respectively. The cell culture supernatant was collected to measure the concentration of inflammatory factor by enzyme-linked immunosorbent assay (ELISA). Macrophages were harvested for flow cytometry and RNA extraction.

### Enzyme-linked immunosorbent assay

The assay kits for measurements of interleukin (IL)-10, prostaglandin E2 (PGE2), and tumor necrosis factor (TNF)-α were purchased from Shanghai Jianglai Biotech, China. Detailed operating steps are according to the products’ instructions. Each sample was measured in triplicate.

### RNA extraction and real-time quantitative PCR

Total RNA was extracted using Trizol reagent (Invitrogen Inc., USA), according to the products’ instructions. The RNA was quantified by measuring the absorbance at 260 nm and 280 nm using a spectrophotometer (NanoDrop Technologies, USA). Real-time quantitative PCR (qRT-PCR) was performed with a CFX96 Real-Time PCR Detection System (Bio-Rad, USA) using one step SYBR PrimeScript RT-PCR Kit (TaKaRa, Japan). Each sample was performed in triplicate. The primers used are shown in Additional file 1: Table S1.

### Flow cytometry analysis

Phenotype of MSCs was analyzed using the following antibodies: fluorescein isothiocyanate (FITC)-conjugated CD73, CD90, CD105, CD44, CD14, CD34, and HLA-DR; phycoerythrin (PE)-conjugated CD106, CD166, CD45, and CD19; and allophycocyanin (APC)-conjugated CD54 (Biolegend, USA). Polarized phenotype of macrophages was analyzed using the following antibodies: FITC-conjugated CD163 (Bio-Rad, USA), PE-conjugated CD86 (BD Biosciences, USA), and Alexa-Flour647-conjugated CD68 (Bio-Rad, USA). Non-specific isotype-matched antibodies served as controls. The cells were analyzed using a flow cytometry instrument (BD CantoII, USA), and the data were analyzed using FlowJo V10 (Tree Star Inc., USA).

### Trilineage differentiation assay

Mesenchymal stem cell adipogenic, osteogenic, or chondrogenic induction medium (Cyagen, Suzhou, China) was used to induce differentiation of MSCs into adipocytes, osteoblasts, and chondrocytes, respectively. To assess adipogenic differentiation, lipid droplets of differentiated cells were stained using Oil Red O. To assess osteogenic differentiation, cells were stained with Alizarin Red S. To assess chondrogenic differentiation, cells were stained with Alcian blue.

### XY chromosome fluorescence in situ hybridization (XY-FISH)

MSCs derived from the placental chorionic plate, chorionic villi, decidua, and umbilical cord were used for FISH assays to identify the ratio of fetal-derived MSCs to maternal-derived MSCs. FISH was carried out using FISH CEP probe of human chromosome X-Green/Y-Orange (GeneCopoeia Inc., USA) according to the manufacturer’s instructions. Laser confocal microscopy (Nikon A1R+, Japan) was used for image acquisition.

### Growth kinetics analysis

The proliferation of MSCs from P3 to P9 was assessed. All MSCs were inoculated on a six-well culture plate at a density of 8 × 10^4^cells/well, and the cells were counted until they reached 100% confluence. The population doubling time (PDT) was calculated using the following formula: PDT = (CT × ln2)/ln (*N*_f_/*N*_i_), where CT is the cell culture time, *N*_i_ is the initial number of cells, and *N*_f_ is the final number of cells [25].

### Cell Counting Kit-8 (CCK-8) assay

The proliferation of MSCs (P4) was determined using the Cell Counting Kit-8 (CCK-8, Dojindo Molecular Technology, Japan). MSCs were adjusted to a concentration of 2 × 10^4^ cells/ml with MSC Serum-Free Medium, and then 100 μl of it was added to each well of the 96-well culture plate. After incubation for the first 24 h, the viable cell number was then tested every 24 h for seven consecutive days. Before the test, 10 μl CCK-8 reagent was added to each well, and the plates were incubated at 37 °C for 2 h. To determine the number of viable MSCs, the optical density value at 450 nm was detected with a spectrophotometer (Multiskan GO, Thermo Scientific).

### Cell cycle analysis

Cell cycle analysis of MSCs was carried out at P4. The cell concentration was adjusted to 1 × 10^6^ cells/ml. After centrifugation, the supernatant was removed and 500 μl 70% cold ethanol was added to the cells for fixation (2 h to overnight), stored at 4 °C. Five hundred microliters of PI/RNase A staining solution was added to the cells after they have been washed with PBS and centrifuged to remove fixing solution. Undergoing incubation for 30 min at 4 °C, the cells were then analyzed using a flow cytometry instrument (BD CantoII, USA) and the data were analyzed using FlowJo V10 (Tree Star Inc., USA).

### Scratch test

Before inoculation of cells, use a marker pen to draw three horizontal lines behind the six-well culture plate at intervals of 0.5 cm. Cells (4 × 10^5^/well) were added to each well, and the plates were incubated at 37 °C. When the cells reached 100% confluence, 200-μl tip was used to draw three scratches perpendicular to the back horizontal line. After being washed by PBS and cultured in serum-free DMEM, cells were observed and photographed at 0 h, 6 h, 12 h, and 24 h. The wound size subsequently was measured and analyzed using ImageJ software (Rawak Software, Inc. Germany).

### Transwell migration assay

Besides the scratch test, migration ability of MSCs was also tested by transwell chambers (24-well culture plate, 8-μm pore size). MSCs were adjusted to a concentration of 2 × 10^5^ cells/ml with MSC Serum-Free Medium, and then 100-μl cell suspension was added to the upper chamber of the migration well. To the contrary, MSC Serum-Free Medium and chemokine stromal-derived factor-1 (SDF-1, 100 ng/ml, Millipore) were loaded into the lower chamber. Then, the cells were cultured at 37 °C in a 5% CO2 incubator, and the plates were taken out at 6 h, 12 h, and 24 h for observation. First, the cells on the filter were removed with a cotton swab, then fixed with 4% paraformaldehyde for 30 min, and finally stained with crystal violet (Beyotime, Haimen, China) for 20 min. Cells in five random separate microscope fields were counted by ImageJ software (Rawak Software, Inc. Germany).

### Statistical analysis

Statistics as well as graphical representations were performed using GraphPad Prism™ 7.0 (GraphPad Software Inc., USA). Parametric data are expressed as the means ± SD. Unless otherwise noticed, differences between the two experimental groups were applied using an unpaired two-tailed Student’s *t* test. For comparison of more than three groups, one-way analysis of variance (ANOVA) was applied, and nonparametrically distributed variables were compared by the Mann-Whitney *U* test using SPSS 18.0 (SPSS Inc., USA). Results were considered statistically significant with *p* values: *p < 0.05, **p < 0.01, and ***p < 0.001.

## Results

### Establishment of an effective isolation and culture system for MSCs

To investigate whether the MEC method is an efficient and suitable method to isolate MSCs from perinatal tissues in SFM, we compared its efficiencies and cell yield with that using the EC method. CP-MSCs and UC-MSCs were taken as representative examples for the comparison of MSC isolate methods. Additionally, cell quality and proliferation were also evaluated.

#### MEC achieves a higher efficiency to isolate MSCs from perinatal tissues in SFM

**Fig. 2:**
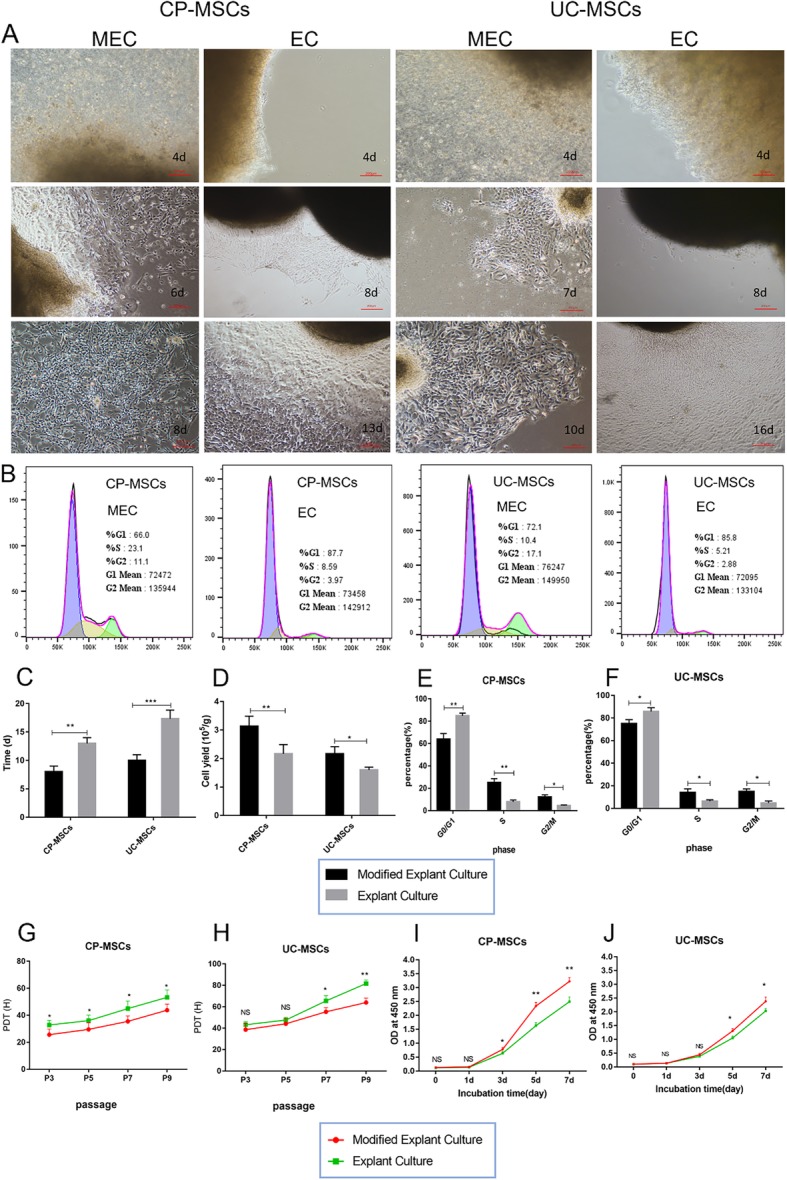
The MEC method is superior to the EC method in terms of efficiency and proliferative properties. **a** Representative diagrams of CP-MSCs and UC-MSCs during MEC and EC (100-fold magnification). **b** Cell cycle diagram of MSCs obtained by MEC and EC. **c** The mean time to the first passage of CP-MSCs and UC-MSCs in the MEC and EC groups. **d** The mean cell yield for the first passage of CP-MSCs and UC-MSCs in the MEC and EC groups. Statistical charts of cell cycle analysis of CP-MSCs (**e**) and UC-MSCs (**f**) obtained by MEC and EC (*N* = 3, P4). PDT of CP-MSCs (**g**) and UC-MSCs (**h**) obtained by MEC and EC (*N* = 3, *n* = 3). CCK-8 assay of CP-MSCs (**i**) and UC-MSCs (**j**) obtained by MEC and EC (*N* = 3, *n* = 6, P4). All data are expressed as the means ± SD (*p < 0.05, **p < 0.01, ***p < 0.001, NS, no significant). MEC, modified explant culture; EC, explant culture; PDT, population doubling time

We simultaneously isolated MSCs from the CP and UC tissue by the MEC and EC methods. At day 4, rod-like and irregularly shaped cells were observed to migrate from the CP and UC tissue in the MEC group while no cells were detected in the EC group. Notably, cells reached approximately 80% confluence in the CP tissue at day 8 and in the UC tissue at day 10 in the MEC group. In contrast, only a few migrated cells were observed at day 8 and cell confluence was detected at days 13 and 16 for the CP and UC tissue respectively in the EC group (Fig. 2a). As shown in Fig. 2c, primary cell confluence took significantly less time with the MEC method than with the EC method. Importantly, the MEC method yielded significantly more cells in comparison with the EC method (Fig. 2d). Overall, these data suggest that the MEC method could be used as an efficient technique to isolate MSCs from perinatal tissues and harvest a higher yield.

#### MSCs obtained by MEC exhibited superior proliferative properties

To determine whether our developed MEC method has any influence on MSC growth, we compared the proliferation rate of MSCs isolated using MEC than that using EC by population doubling time (PDT), CCK-8 assay, and cell cycle analysis. PDT of MSCs obtained by MEC was significantly shorter than that of MSCs obtained by the EC method (Fig. 2g, h). The CCK-8 assay also showed that MSCs obtained by the MEC method had stronger proliferative activity (Fig. 2i, j). This result was further supported by the cell cycle analysis in which the MEC method had more cells in the dividing phase (Fig. 2b, e, f).

### Advantageous properties of the generated CP-MSCs using the new protocol

To determine whether the biological properties of P-MSCs using MEC is related to the anatomical sites of the placenta, we thus simultaneously isolated from the different regions of the placenta using the new protocol, including CP-MSCs, CV-MSCs, and D-MSCs. Here, UC-MSCs were used as a control. The quality of these various P-MSCs was assessed to verify their function properties.

#### MSCs obtained by MEC meet the criteria of MSCs proposed by the ISCT

To determine whether the isolated MSCs using MEC were truly MSCs, we identified the biological characteristics of all isolated MSCs by immunophenotypic analysis and trilineage differentiation assay.

**Fig. 3:**
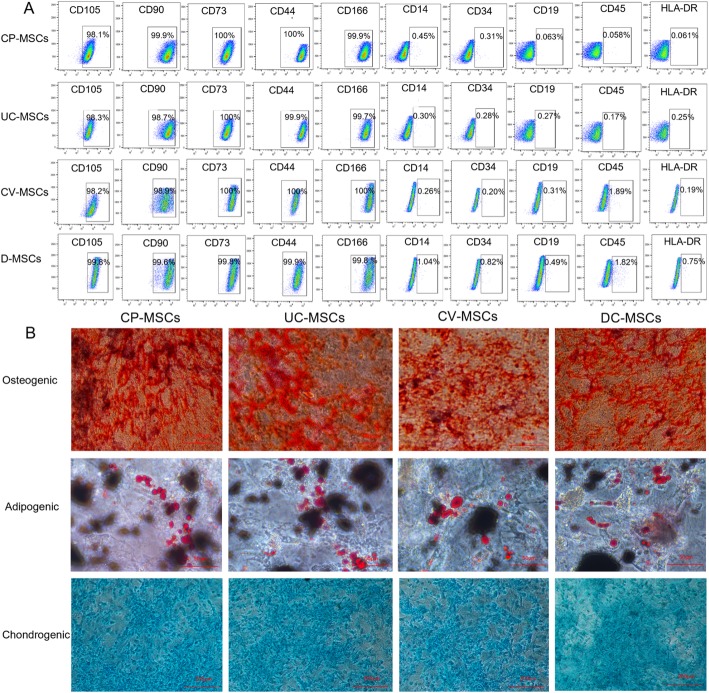
MSCs obtained by MEC meet the criteria of MSCs proposed by the ISCT. **a** Cell surface markers of MSCs (*N* = 3, P3) were analyzed by flow cytometry. All MSCs were positive for CD105, CD90, CD73, CD44, and CD166 (≥ 95%) and negative for CD14, CD34, CD19, CD45, and HLA-DR (≤ 2%). **b** Trilineage differentiation of all MSCs (P3)

Immunophenotypic analysis, regardless of differences in origin, of all MSCs exhibited a characteristic marker profile. MSCs were positive for CD73, CD90, CD105, CD44, and CD166 (≥ 95%) and negative for CD45, CD34, CD14, CD19, and HLA-DR (≤ 2%) (Fig. 3a, Additional file 1: Table S2). In vitro differentiation assay showed that all MSCs were able to differentiate into adipogenic, osteogenic, and chondrogenic lineages (Fig. 3b). The above data indicates that all MSCs meet the criteria of MSCs proposed by the International Society for Cellular Therapies (ISCT) [26].

#### CP-MSCs are of fetal origin and superior to other P-MSCs in the origins

To identify the ratio of fetal-derived MSCs to maternal-derived MSCs, the isolated MSCs including CP-MSCs, CV-MSCs, D-MSCs, and UC-MSCs were used for XY chromosome fluorescence in situ hybridization (XY-FISH) experiments.

**Fig. 4:**
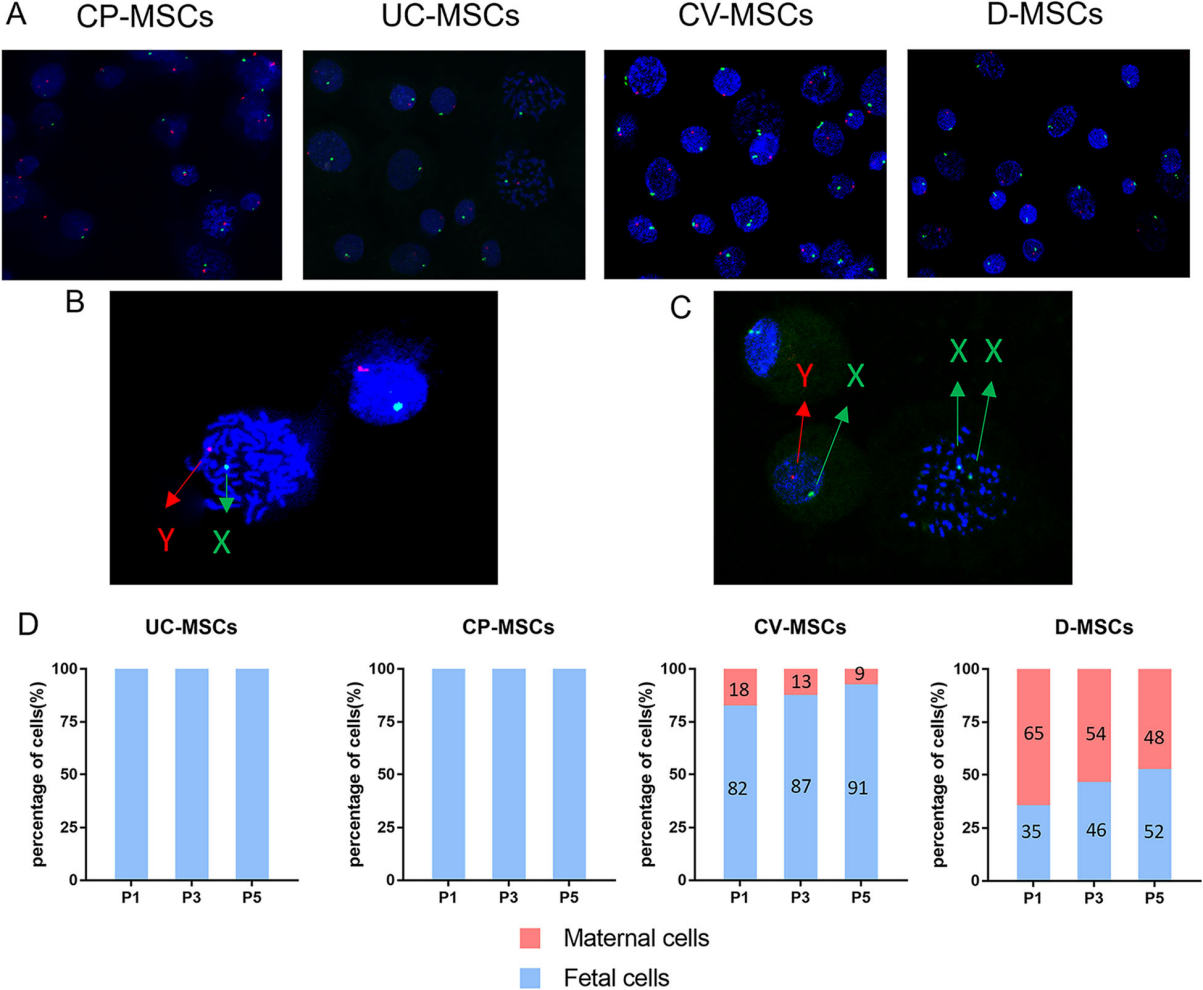
CP-MSCs are of fetal origin and superior to other P-MSCs in the origins. **a** XY-FISH plots of MSCs (green dots represent X chromosome, red dots represent Y chromosome, P3). **b** Magnified view of FISH results from CP-MSCs. **c** Magnified view of FISH results from CV-MSCs. **d** Quantitative determination of fetal (male) and maternal (female) cells during ex vivo expansion of all MSCs using XY-FISH. The *X*-axis represents passage numbers, and the *Y*-axis represents the percentage of XY male (blue) or XX female (red)

As shown, the fetal-derived MSCs appear as one X chromosome (green) and one Y chromosome (red) while the maternal-derived MSCs appear as two X chromosomes (Fig. 4a–c). No maternal-derived MSCs were noted in CP-MSCs and UC-MSCs from P1 to P5. However, the proportion of maternal-derived MSCs was 18 ± 7%, 13 ± 6%, and 9 ± 4% in P1, P3, and P5 of CV-MSCs respectively and 65 ± 15%, 54 ± 11%, 48 ± 10% respectively in P1, P3, and P5 of D-MSCs (Fig. 4d). In addition, we found that the proportion of maternal-derived MSCs in CV-MSCs and D-MSCs gradually decreased with the increase of culture passage. These results suggest that CP-MSCs are superior to other P-MSCs in the origins and that the serum-free culture system we use is more suitable for the proliferation of fetal-derived MSCs.

#### CP-MSCs show higher proliferation ability

**Fig. 5:**
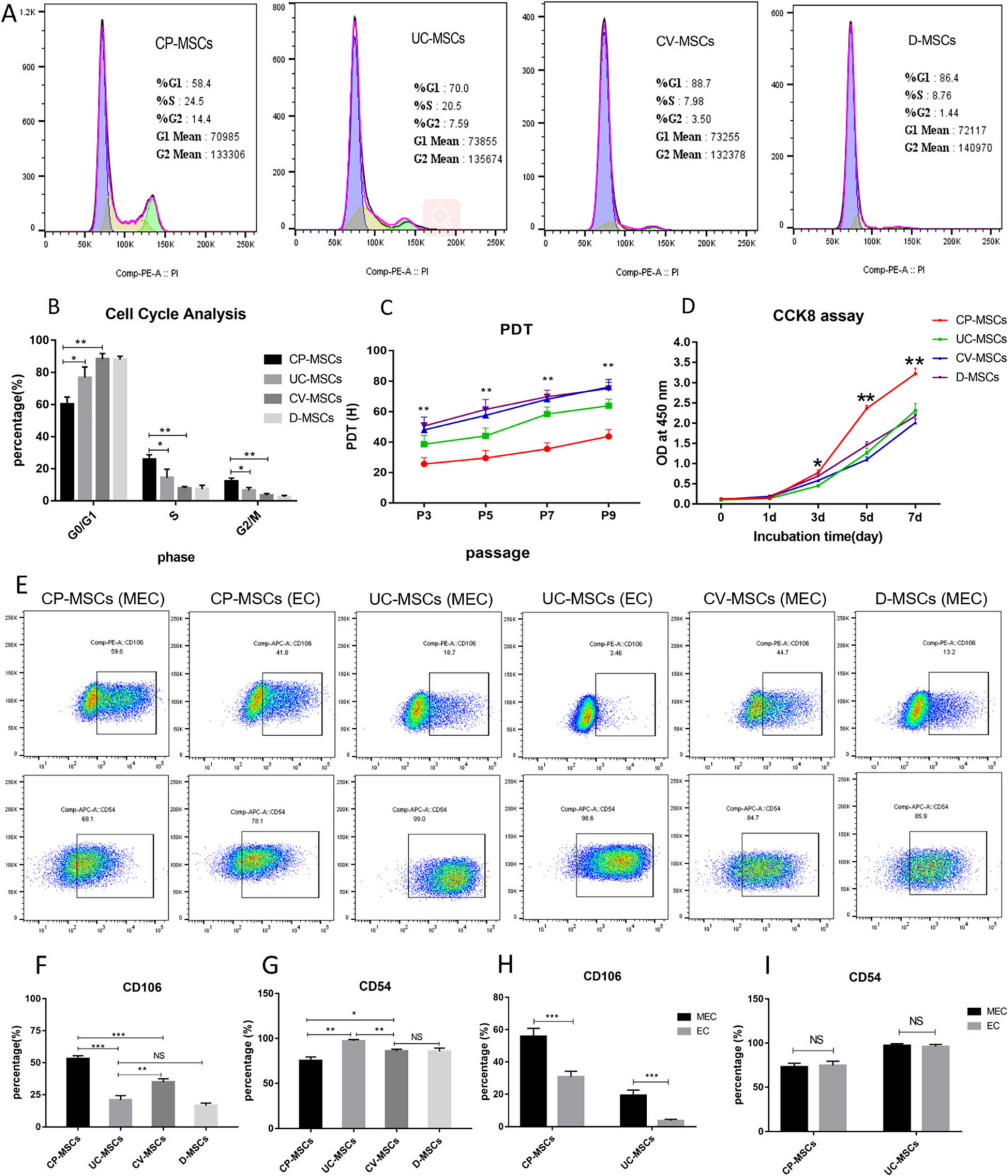
CP-MSCs showed higher proliferation ability. **a** Cell cycle diagram of MSCs. **b** Statistical charts of cell cycle of MSCs. **c** The PDT of MSCs. **d** Growth curves of MSCs. **e** FSC-H profiles of typical MSCs. **f** Statistic analysis of CD106 expression in different MSCs. **g** Statistic analysis of CD54 expression in different MSCs. CD106 (**h**) and CD54 (**i**) of CP-MSCs and UC-MSCs obtained by MEC and EC. All data are expressed as the means ± SD (*N* = 3; *p < 0.05, **p < 0.01, ***p < 0.001, NS, no significant)

As shown in Fig. 5, CP-MSCs, CV-MSCs, D-MSCs, and UC-MSCs from the same donor showed different proliferative capacities at the same culture passage. The PDT of CP-MSCs was significantly shorter than that of the other three MSCs, and PDT of all MSCs gradually prolonged with the increase of culture passage (Fig. 5c). The data indicated that CP-MSCs proliferated the fastest, followed by UC-MSCs, and CV-MSCs and DC-MSCs with similar proliferation ability. To further confirm that CP-MSCs have greater proliferative capacity, all MSCs were then seeded at the same quantity for the CCK-8 assay. The viable cell quantity among them was almost the same at day 1, but started to be significantly different from day 3 (Fig. 5d). The tendency of the growth curve also verified that CP-MSCs possessed higher proliferation ability. The cell cycles of all MSCs from the three donors were assessed at passage 4. Cell cycle analysis shows that CP-MSCs have more cells in the dividing phase than the other three MSCs (Fig. 5a, b).

#### CP-MSCs highly express CD106

Some studies have shown that CD106 and CD54 are involved in MSC-mediated immunomodulation [27], and CD106^+^MSCs have stronger immunomodulatory capacity than CD106^−^MSCs [28]. Therefore, we analyzed the expression of CD106 and CD54 on the surface of these four MSCs. The results showed that CP-MSCs highly expressed CD106 (Fig. 5e, f). The expression of CD54 in MSCs appeared to be opposite to that of CD106. CP-MSCs with high expression of CD106 had low expression of CD54, whereas UC-MSCs with low expression of CD106 highly expressed CD54 (Fig. 5e–g). In addition, we also found that MSCs obtained by the MEC method have higher expression of CD106 than MSCs obtained by EC (Fig. 5e, h).

#### CP-MSCs show stronger migration ability

**Fig. 6:**
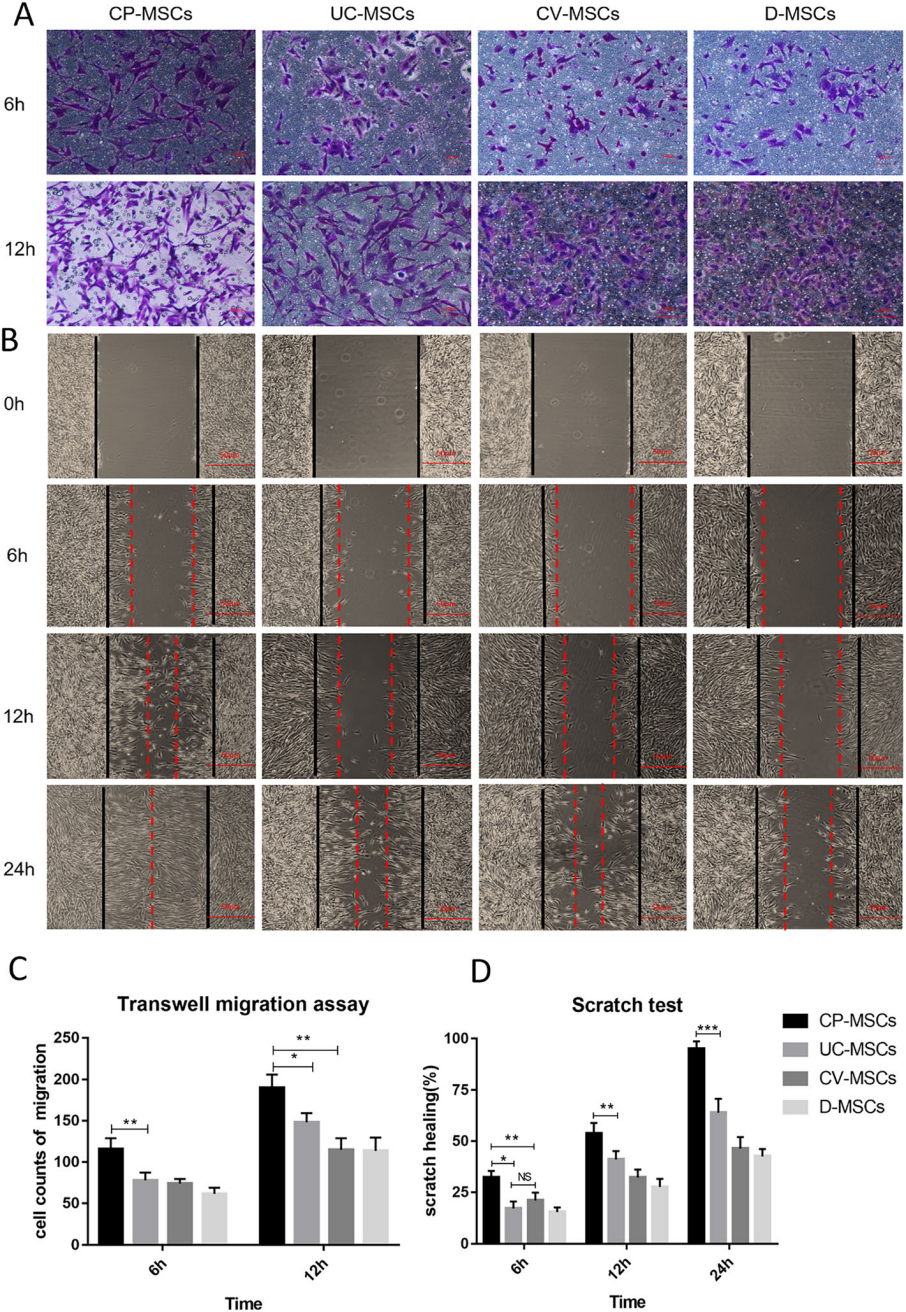
CP-MSCs show stronger migration capability. **a** Representative images of MSC transwell migration assay at 6 h and 12 h (200-fold magnification). **b** Representative figures of MSC scratch test at 0 h, 6 h,12 h, and 24 h (the black line indicates the scratch width at 0 h, and the red dotted line indicates the scratch width at each time point, 40-fold magnification). **c** Cell counts of transwell migration at 6 h and 12 h. **d** Healing area/wounded area ratio in scratch test analysis at 6 h, 12 h, and 24 h. All data are expressed as the means ± SD (*N* = 3, *p < 0.05, **p < 0.01, ***p < 0.001, NS, no significant)

To compare the migration ability of MSCs, all MSCs were subjected to scratch test and transwell migration assay. First, we evaluate MSC migration capacity by introducing scratch test to assess healing area/wounded area ratio at 6 h, 12 h, and 24 h. The figure (Fig. 6b, d) showed that the healing area/injured area ratio of CP-MSCs was higher than that of the other three MSCs. Second, we further compared the migration ability of MSCs by transwell migration assay and crystal violet staining to count the number of migrated cells after 6 h and 12 h of MSC inoculation. The results (Fig. 6a, c) illustrated that the number of migrated cells in the CP-MSC group was significantly higher than that of the other three groups. In the transwell migration assay, we observed that almost all cells penetrated the filter membrane when MSCs were inoculated for 24 h, and some of the cells had grown on the well plates. Since it was difficult to count the number of migrated cells, we did not evaluate their migration ability in the 24-h group. The above results revealed that CP-MSCs possess stronger migration capabilities.

#### CP-MSCs show greater ability to regulate macrophage from M1 to M2

We have found that CP-MSCs highly express CD106 compared to the other three MSCs, and a study has reported that CD106^+^MSCs exhibit stronger immunomodulatory capacity than CD106^−^MSCs. In order to compare the immunomodulatory ability of MSCs from different regions of the placenta to macrophage polarization, we co-cultured MSCs with macrophages via transwell chamber.

**Fig. 7:**
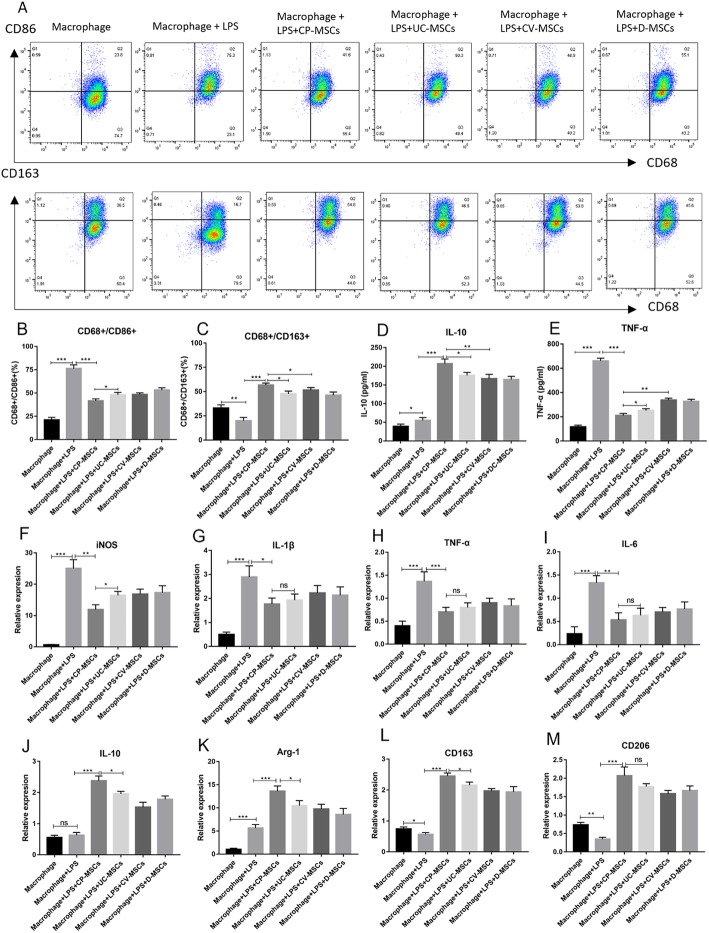
CP-MSCs show greater ability to regulate macrophage from M1 to M2. **a**–**c** Flow cytometry analysis of macrophage polarization, M1 polarization marker (CD86), M2 polarization marker (CD163). **d**, **e** The concentration of TNF-a and IL-10 in the cell culture supernatant. **f**–**m** The expression of major M1 markers (IL-1β, TNF-α, iNOS, and IL-6) and M2 markers (IL-10, Arg-1, CD163, and CD206). All data are expressed as the means ± SD (*N* = 3; *p < 0.05, **p < 0.01, ***p < 0.001, NS, no significant)

Flow cytometry analysis revealed that only a few cells expressed CD86 and CD163 in macrophages that were not stimulated by LPS. After LPS stimulation, the number of cells expressing CD86 was significantly increased, and the number of cells expressing CD163 was decreased. However, after co-culture with MSCs, the number of cells expressing CD86 was significantly decrease, while the number of cells expressing CD163 increased significantly. After co-culture with MSCs, CD68^+^CD86^+^ macrophages in the CP-MSC group were lower than other three groups, while CD68^+^CD163^+^macrophages in the CP-MSC group were higher than other three groups (Fig. 7a–c). Flow cytometry analysis preliminary showed that CP-MSCs showed stronger immunomodulatory ability than UC-MSCs, CV-MSCs, and D-MSCs.

ELISA was used to determine the concentration of inflammatory factors in the cell culture supernatant. The concentration of TNF-α significantly increased after LPS of administration, whereas that dramatically decreased after co-culture with MSCs. Moreover, the concentration of TNF-α was the lowest in the CP-MSC group. The concentration of IL-10 slightly increased after LPS intervention, while that remarkably increased after co-culture with MSCs. In contrast, IL-10 was the richest in the CP-MSC group (Fig. 7d, e).

To further confirm that CP-MSCs exhibit stronger immunomodulatory capacity in regulating macrophage polarization, the expression of major M1 markers (IL-1β, TNF-α, inducible nitric oxide synthase (iNOS), and IL-6) and M2 markers (IL-10, arginase (Arg)-1, CD163, and CD206) were evaluated using qRT-PCR. As shown in Fig. 7f–m, the results indicated that after macrophage stimulation by LPS, the gene expression of M1 marker was significantly increased, and the gene expression of CD163 and CD206 were significantly decreased, while the gene expression of Arg-1 was significantly elevated. However, after co-culture with MSCs, the gene expression of the M1 marker was significantly lower than that of the LPS group, while the gene expression of the M2 marker was significantly increased, and CP-MSCs show greater immunomodulatory capacity compared with the other three MSCs. Taken together, these results suggest CP-MSCs exhibit stronger ability to regulate macrophage polarization.

## Discussion

In the present study, we developed an efficient and high-yield technique to produce high-quality MSCs from the placenta, which is suitable for clinical application. For this technique, the MEC method, serum-free culture system, and the selection of the CP are critical requirements for successfully generating large numbers of pure fetal P-MSCs. The important findings of this study are as follows: (i) The MEC method achieves higher cell yield and shorter time in primary cell confluence in SFM. The harvested cells are found to show stronger proliferation ability. (ii) The serum-free culture system we used is more suitable for fetal cell growth and has enrichment effect on fetal MSCs; and (iii) CP seems to be preferable to the tissue-harvesting site for generating high-quality MSCs. These CP-MSCs exhibit superior properties in proliferation, migration, and immunomodulatory properties, maintaining the fetal origin over serial passages.

Choosing a suitable isolation method is a vital step in generating P-MSCs with optimal quality and high yield to meet the clinical demand. To date, there are two main methods for isolating P-MSCs including the enzymatic method and the EC method. However, there are some problems regarding the two methods. Enzymatic methods are reported to affect the biological characteristics of MSCs such as proliferative capacity [19, 20]. In addition, enzymatic methods are more complicated, and the operation process will take more time and money. On the other hand, the EC method requires a longer time for obtaining cells in primary culture than enzymatic methods, which is due to the slower rate of migration of cells from the tissue block. To overcome the above limitations, we developed the MEC method for isolation and culture of P-MSCs by combining an initial mild enzymatic reaction with the subsequent explant culture. The obtained MSCs by MEC possessed MSC characteristics and had stronger proliferation ability than those with the EC method. Notably, the MEC method was found to have the following advantages over the EC method: (i) cells can migrate out of the tissue block in a shorter time; (ii) the cell yield is higher and more MSCs can be obtained in a shorter time; and (iii) MSCs show stronger proliferative activity. These results confirm that the MEC method can shorten the primary confluence time and generate large numbers of P-MSCs, indicating the MEC method is an effective and high-yield technique to isolate P-MSCs from the placenta.

In addition to large quantities of MSCs, as required for clinical therapies, optimal quality is another crucial factor. A series of studies showed that fetal-derived MSCs isolated from the placenta have stronger proliferation and differentiation ability, immune regulation, angiogenesis, and migration ability than maternal-derived MSCs [13, 17, 29, 30]. Thus, researchers are trying to obtain the pure fetal P-MSCs for clinical applications. However, maternal cell contamination remains a genuine problem during P-MSC isolation and culture. For this reason, the variation in MSCs obtained from different regions of the placenta is one key determinant. Considering the completely fetal origin of CP tissues [21], we thus selected CP as the tissue-harvesting site for generating optimal quality of P-MSC. As evidenced by XY-FISH experiments, our results showed CP-MSCs (P1-P5) were absent of maternal cells, indicating that the obtained CP-MSCs are pure fetal cells without maternal cell contamination. Thus, the specific selection of CP is one key requirement for the successful isolation and expansion of pure fetal P-MSCs.

For generating pure fetal P-MSCs, the serum-free culture system is another critical requirement. To date, P-MSCs that are almost isolated and expanded in the serum-containing medium (SCM) are reported to appear less and less of fetal cells with the prolongation of culture time even though there is a large number of fetal cells in the early stage [16, 18, 21]. Given the complex and unclear composition, SCM might bring various uncertainties to the results of MSC therapy, thereby hindering the large-scale clinical use of MSCs [1, 31]. In this study, we used SFM to isolate and culture MSCs, which has no serum and no animal source components, and clear chemical components. Our result demonstrated that P-MSCs exhibited good biological properties in terms of proliferation, differentiation migration, and immunomodulatory properties in SFM culture, suggesting that our used SFM is suitable for the isolation and expansion of MSCs for clinical application. The result is consistent with the previous report illustrating the advantageous properties of MSC cultured in SFM relative to SCM [32, 33]. Additionally, using the SFM, CV-MSCs contained a small number of maternal cells in primary culture, while D-MSCs contained a large number of maternal cells in primary culture. However, we found that maternal cells gradually decreased while fetal cells gradually increased over serial passages. These data suggest that the serum-free culture system is not conducive to the proliferation of maternal MSCs. Overall, our used serum-free culture system is suitable for isolating and expanding pure fetal MSCs for clinical therapy.

Macrophages are plastic cells displaying versatile functional phenotypes that are dependent on microenvironments [34]. M1 macrophages (M1, the classically activated macrophages) and M2 macrophages (M2, the alternatively activated macrophages) have been defined as the two extremes in a spectrum of macrophage functional phenotypes [35]. The former are mainly involved in pro-inflammatory, and phagocytic response that effectively clears pathogens [36], while the latter mainly participates in anti-inflammatory response and promotes angiogenesis, tissue repair, and wound healing [37]. Numerous studies have confirmed that MSCs can regulate macrophage polarization from M1 to M2 by secreting prostaglandin E2 (PGE2) [38], TNF-α-induced gene/protein 6 (TSG-6) [39], transforming growth factor (TGF) β3 [40], thrombospondin-1 (TSP-1) [40], indoleamine 2,3-dioxygenase (IDO) [41], exosomes [42], and extracellular vesicle mitochondrial transfer [43]. In this study, we found that CP-MSCs showed stronger immunomodulatory ability in regulating macrophage polarization from M1 to M2 when compared to UC-MSCs and other P-MSCs. These data suggest CP-MSCs may be optimal source for clinical treatment of immune diseases. In addition, our preliminary study found that CP-MSCs highly expressed COX-2, while PGE-2 in CP-MSC culture supernatant was higher than that of the other three MSCs (Additional file 1: Figure S2). However, the detailed mechanism by which CP-MSCs exhibit stronger immunomodulatory ability than the other three MSCs is still unclear, and more work are needed to be done in our future study.

CD106, known as vascular cell adhesion molecule-1 (VCAM-1), is extensively expressed on endothelial cells and is also constitutively expressed on some stromal cells. Recently, some studies have found that CD106 is expressed in a fraction of MSCs, varying from 30 to 75% in human BM-MSCs and P-MSCs, and lower expression in UC-MSC and adipose tissue-derived MSCs (AT-MSC) [23, 25, 44]. Cell–cell adhesion mediated by CD106 is critical for T cell activation and leukocyte recruitment to the inflammation site and, therefore, plays an important role in evoking effective immune responses. Ren et al. showed that CD106 plays an important role in MSC-mediated immunosuppression [27]. Yang and colleagues demonstrated that CD106^+^MSCs have stronger immunomodulatory ability than CD106^−^MSCs [28]. In this study, we found that CP-MSCs highly expressed CD106 compared to UC-MSCs, CV-MSCs, and D-MSCs. This result is consistent with our above finding that CP-MSCs have better immunomodulatory effect on M2 macrophage polarization.

Additionally, a series of studies have shown that MSCs can regulate the activation of various immune cells by secreting various cytokines (such as PGE2, IDO, HGF, NO, and exosomes), including T lymphocytes, B lymphocytes, dendritic cells, natural killing cells, and macrophages [3]. Hence, MSCs have been widely used in the therapeutic research of some immune diseases. Interestingly, MSCs have shown to be of crucial importance for the pregnancy and reception of the embryo. It is reported that bone mesenchymal stem cells improve pregnancy outcome by inducing maternal tolerance to the allogeneic fetus in abortion-prone matings in mouse [45]. Another study shows that MSCs have a therapeutic effect in an immune-based mouse model of recurrent spontaneous abortion, which may be relevant in the decidual mechanisms of maternal–fetal immune tolerance [46]. Both studies indicate MSCs possess the ability to improve maternal–fetal immune tolerance. However, whether CP-MSCs are involved in the modulation of maternal–fetal immune tolerance is not known, and we will focus on this issue in the future.

## Conclusion

In conclusion, our study developed an efficient and high-yield technique to produce high-quality MSCs from the placenta. Moreover, our results presented that the harvested CP-MSCs exhibit superior proliferative, migration, and immunomodulatory properties, hence serving as an optimal source of MSCs for clinical application.

## Supplementary information

**Additional file 1: Table S1.** Primer used for real-time quantitative PCR. **Table S2.** Immunophenotyping of cells derived from various sources by flow cytometry. **Figure S1.** Growth status of MSCs derived from different regions of the umbilical cord and placenta. **Figure S2.** COX-2 and PGE2 of MSCs from different tissues. **Figure S3.** CP-MSCs are the best choice for P-MSCs isolation. (DOCX 1182 kb)

## Data Availability

All data generated or analyzed during this study are included in this published article.

## Abbreviations

AM: Amniotic membrane
ANOVA: Analysis of variance
APC: Allophycocyanin
Arg-1: Arginase-1
AT-MSCs: Adipose tissue-derived MSCs
BM: Bone marrow
CCK-8: Cell Counting Kit-8
CP: Chorionic plate
CP-MSCs: Chorionic plate-derived MSCs
CV: Chorionic villi
CV-MSCs: Chorionic villi-derived MSCs
D: Decidua
DMEM: Dulbecco’s modified Eagle medium
D-MSCs: Decidua-derived MSCs
EC: Explant culture
ELISA: Enzyme-linked immunosorbent assay
FISH: Fluorescence in situ hybridization
FITC: Fluorescein isothiocyanate
GVHD: Graft versus host disease
IDO: Indoleamine 2,3-dioxygenase
IL: Interleukin
iNOS: Inducible nitric oxide synthase
ISCT: International Society for Cellular Therapies
LPS: Lipopolysaccharide
MEC: Modified explant culture
MSCs: Mesenchymal stem cells
P: Placenta
PBS: Phosphate-buffered saline
PDT: Population doubling time
PE: Phycoerythrin
PGE2: Prostaglandin E2
P-MSCs: Placenta-derived MSCs
SCM: Serum-containing medium
SDF-1: Stromal-derived factor-1
SFM: Serum-free medium
TGF: Transforming growth factor
TNF-α: Tumor necrosis factor-α
TSG-6: TNF-α-induced gene/protein 6
TSP-1: Thrombospondin-1
UC: Umbilical cord
UC-MSCs: Umbilical cord-derived MSCs
VCAM-1: Vascular cell adhesion molecule-1
