# Supplementary material for: An efficient protocol to generate placental chorionic plate-derived mesenchymal stem cells with superior proliferative and immunomodulatory properties
Source: Stem Cell Res Ther. 2019 Oct 17;10:301. doi: 10.1186/s13287-019-1405-8 (PMC6796371; doi:10.1186/s13287-019-1405-8)
Supplement: Supplementary file 1 — Additional file 1: Table S1. Primer used for real-time quantitative PCR. Table S2. Immunophenotyping of cells derived from various sources by flow cytometry. Figure S1. Growth status of MSCs derived from different regions of the umbilical cord and placenta. Figure S2. COX-2 and PGE2 of MSCs from different tissues. Figure S3. CP-MSCs are the best choice for P-MSCs isolation. (DOCX 1182 kb) [file 13287_2019_1405_MOESM1_ESM.docx]

**Supplementary Material**

**An efficient protocol to generate placental chorionic plate-derived mesenchymal stem cells with superior proliferative and immunomodulatory properties**

Qilin Huang, Yi Yang, Chen Luo, Yi Wen, Ruohong Liu, Shuai Li, Tao Chen, Hongyu Sun, and Lijun Tang

**The subculture of MSCs**

From the passage 1(P1), it needs to be subcultured when the cell confluence reaches 80%-90%. After MSCs are digested by Stem Cell Mild Digestive Enzyme (Yocon, China) for 2 minutes, add 5 times the volume of PBS to the added digestive enzyme, then transfer the cell suspension to the centrifuge tube, centrifuge (1000 rpm, 5 min, 4℃). The pellet was resuspended with MSC Serum Free Media, inoculated into the new culture flask at a density of 8000/cm2. Generally, the cells can grow up to 80%-90% of the culture flask for 72 hours, and the cell culture medium is replaced every 2 days. Serum-Free Cell Cryopreservation Solutions (Yocon, China) was used for cell cryopreservation.

**Isolation of macrophages**

Macrophages were isolated as described earlier. Briefly, after anesthesia with isoflurane, the rats were cut through the abdominal line and intraperitoneally injected with 20ml RPMI-1640 serum-free medium containing 1% penicillin-streptomycin. Then the abdominal cavity was closed and the abdomen was gently massaged for 2 min. Followed by, the intraperitoneal fluid was transferred to a centrifuge tube and centrifuged (300g, 5min, 4°C). The pellet was resuspended in RPMI-1640 medium (Hyclone, USA) containing 10% fetal bovine serum (FBS; Gibco, USA) and inoculated into a six-well culture plate, which was cultured at 37℃ in a humidified atmosphere with 5% CO2. After 2h, the non-adherent cells were removed by changing the medium, and the adherent cells were further cultured for subsequent experiments.

Table S1. Primer used for real-time quantitative PCR.

| mRNA | Forward sequence (5^’^-3’) |  | Reverse sequence (5^’^-3’) |
| --- | --- | --- | --- |
| TNF-α | CGTCGTAGCAAACCACCAAG |  | CACAGAGCAATGACTCCAAAG |
| iNOS | CAGCCCTCAGAGTACAACGAT |  | CAGCAGGCACACGCAATGAT |
| IL-6 | CCCACCAGGAACGAAAGTCA |  | AGTCCCAAGAAGGCAACTGG |
| IL-1β | CCCTGAACTCAACTGTGAAATAGCA |  | CCCAAGTCAAGGGCTTGGAA |
| IL-10 | CAGACCCACATGCTCCGAGA |  | CAAGGCTTGGCAACCCAAGTA |
| Arg-1 | ATCGGAGCGCCTTTCTCTAA’ |  | AGACCGTGGGTTCTTCACAA |
| CD163 | CAACCGATGCTCAGGAAGAG’ |  | GATGGCACTTCCACATCCAA |
| CD206 | ATTCCGGTCGCTGTTCAACT |  | AACGGAGATGGCGCTTAGAG |
| COX-2 | ACTCTGGCTAGACAGCGTAA |  | ACCGTAGATGCTCAGGGAC |
| GAPDH | GTATGACTCTACCCACGGCAAGT |  | TTCCCGTTGATGACCAGCTT’ |

TNF-α, tumor necrosis factor-α; iNOS, inducible nitric oxide synthase; IL, interleukin; Arg-1, arginase-1; COX-2, Cyclooxygenase-2; GAPDH, glyceraldehyde 3-phosphate dehydrogenase.

Table S2. Immunophenotyping of cells derived from various sources by flow cytometry.

|  | CP-MSCs | UC-MSCs | CV- MSCs | D-MSCs |
| --- | --- | --- | --- | --- |
| CD105 | 97.5±1.0 | 97.7±1.2 | 97.3±1.1 | 98.5±1.3 |
| CD90 | 99.5±0.3 | 98.2±0.6 | 98.2±0.7 | 98.8±1.0 |
| CD73 | 99.4±0.4 | 99.1±0.6 | 99.2±0.5 | 98.7±1.2 |
| CD166 | 99.7±0.2 | 99.2±0.5 | 99.4±0.5 | 98.5±1.3 |
| CD44 | 99.6±0.3 | 99.3±0.6 | 99.3±0.4 | 98.1±1.7 |
| CD45 | 0.2±0.1 | 0.2±0.2 | 1.1±0.8 | 1.5±0.5 |
| CD19 | 0.1±0.1 | 0.3±0.2 | 0.3±0.2 | 0.3±0.3 |
| CD34 | 0.3±0.2 | 0.2±0.1 | 0.2±0.1 | 0.5±0.4 |
| CD14 | 0.3±0.2 | 0.2±0.2 | 0.3±0.2 | 0.7±0.4 |
| HLA-DR | 0.1±0.1 | 0.3±0.1 | 0.2±0.1 | 0.7±0.6 |

Results expressed as the means ± SD of marker expression (N=3, P3). Undifferentiated cells were collected and marked with monoclonal antibodies. CP-MSCs, chorionic plate-derived MSCs; UC-MSCs, umbilical cord-derived MSCs; CV-MSCs, chorionic villi-derived MSCs; D-MSCs, decidua-derived MSCs.

**Growth status of MSCs derived from different regions of the umbilical cord and placenta**

In the D-MSCs group, although a large number of cells migrated at an early stage, they were mainly resembling round cells and fewer spindle cells. In addition, as the culture time prolonged, resembling round cells gradually died and were replaced by spindle cells. In the CV-MSCs group, resembling round cells were also observed in the early stage, but they were significantly less than that of the D-MSCs group, and there were more spindle cells. However, resembling round cells that exist in the early stage of decidua group dramatically disappear in CP-MSCs and UC-MSCs group in the whole culture process. These indicated that MSC Serum Free Media is selective for cell growth.


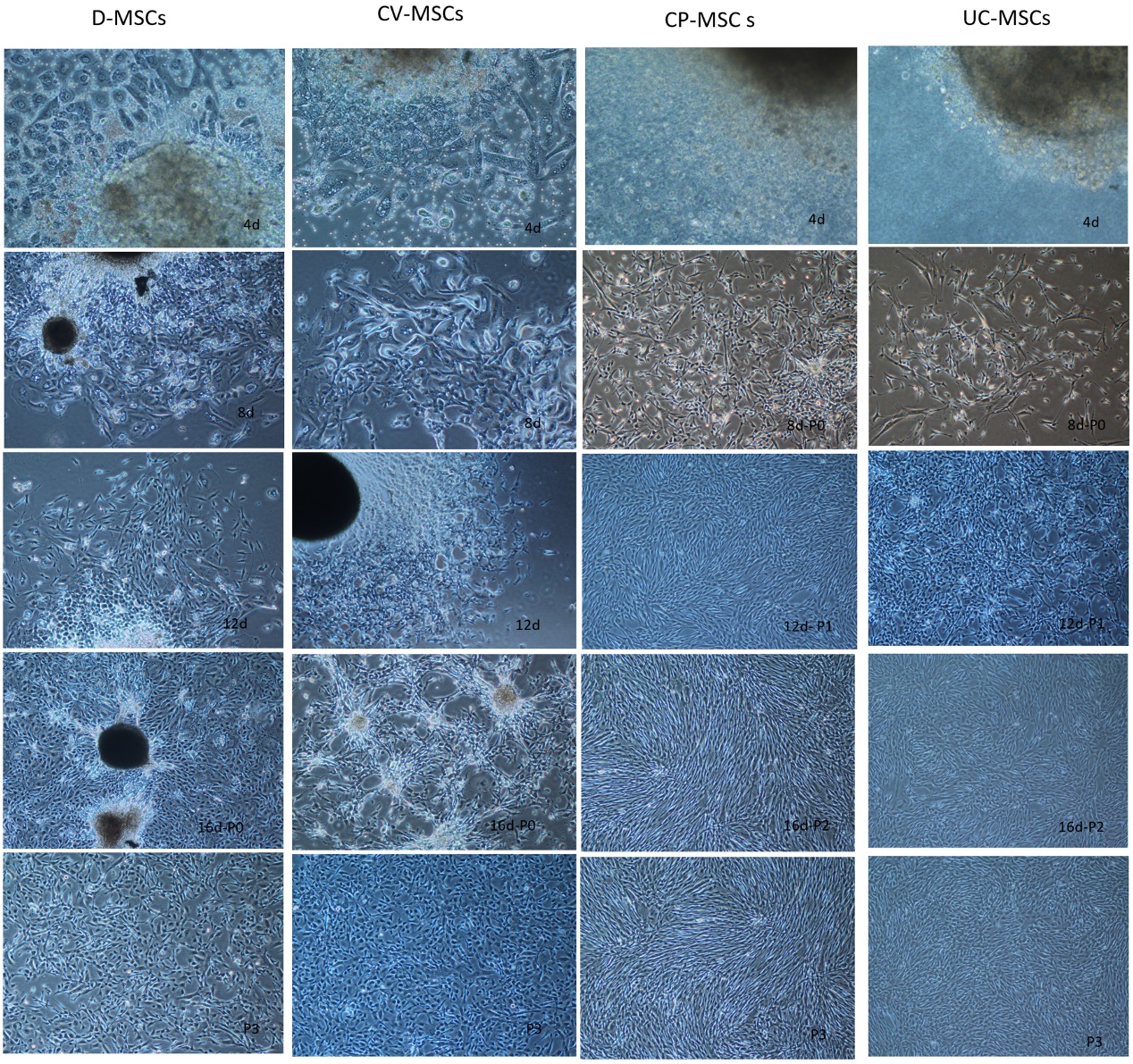


Fig.S1 Growth status of MSCs derived from different regions of the umbilical cord and placenta

Photomicrographs of MSCs from Umbilical cord (UC-MSCs), Chorionic plate (CP-MSCs), Chorionic villi (CV-MSCs) and the decidua (D-MSCs) are shown.


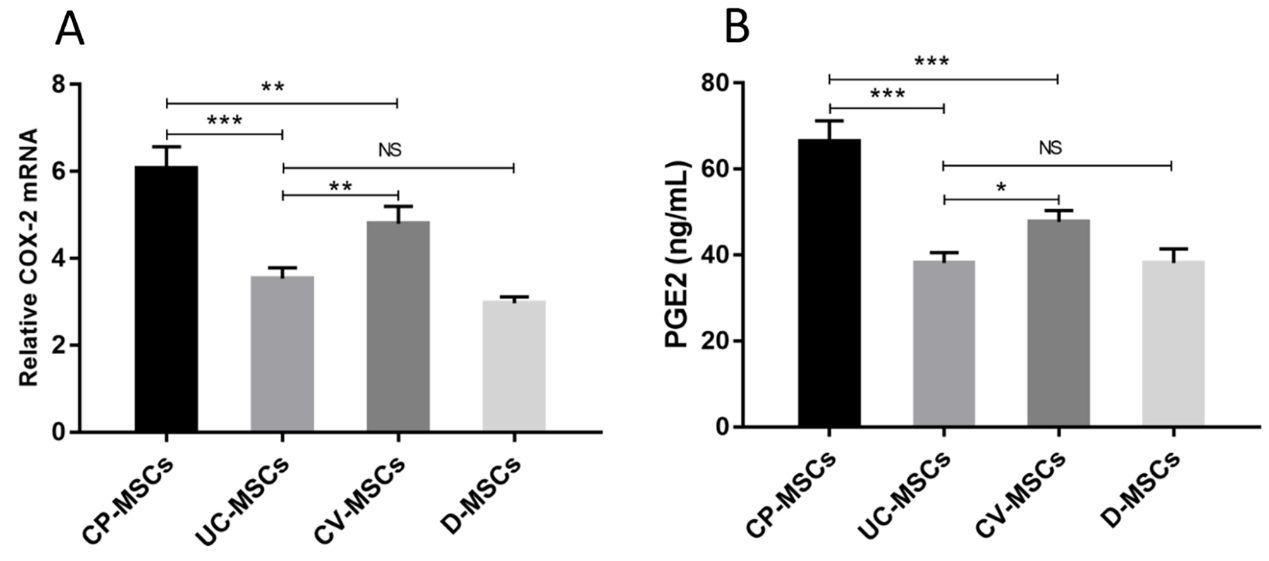


**Fig.S2 COX-2 and PGE2 of MSCs derived from different tissues**

(A) COX-2 gene expression levels of MSCs derived from different tissue. (B) The concentration of PGE2 in culture supernatant of MSCs derived from different tissue. All data are expressed as the means ± SD (N=3; *P < 0.05, **P < 0.01, ***P < 0.001, NS, no significant).

**CP-MSCs are the best choice for P-MSCs isolation**

we found that MSCs from the different regions of placenta had different confusion time of primary cells: CP-MSCs first arrived, followed by UC-MSCs, DC-MSCs and CV-MSCs were latest (Fig.S3A). The yield of MSCs was different for different regions of placenta: CP-MSCs were the highest, followed by UC-MSCs, and CV-MSCs and D-MSCs were the lowest (Fig.S3B).


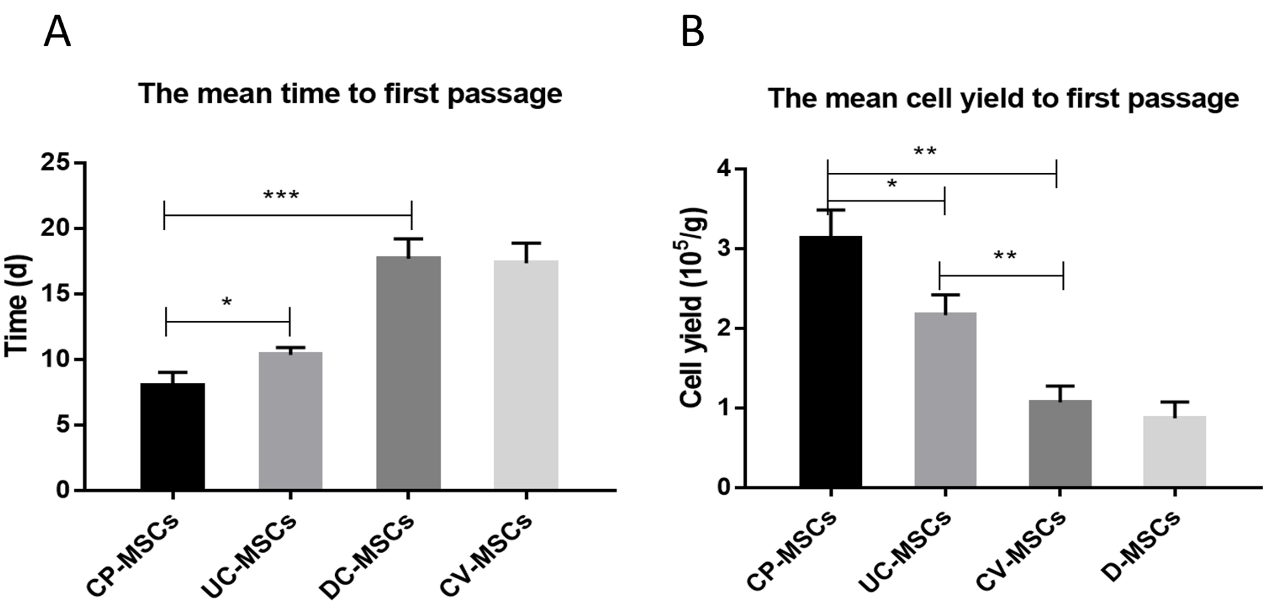


Fig.S3 CP-MSCs are the best choice for P-MSCs isolation

(A) The mean time to first passage of all MSCs. (B) The mean cell yield to first passage of all MSCs. All data are expressed as the means ± SD (N=3; *P < 0.05, **P < 0.01, ***P < 0.001).
